# Retraction Note: Hsa_circ_0001944 promotes the growth and metastasis in bladder cancer cells by acting as a competitive endogenous RNA for miR-548

**DOI:** 10.1186/s13046-023-02602-7

**Published:** 2023-01-24

**Authors:** Mingming Jin, Shengjie Lu, Yue Wu, Chen Yang, Chunzi Shi, Yanqiu Wang, Gang Huang

**Affiliations:** 1 Shanghai University of Traditional Chinese Medicine, Shanghai, 201203 PR China; 2 Shanghai Key Laboratory of Molecular Imaging, Shanghai University of Medicine and Health Sciences, 279 Zhouzhu Road, Pudong New Area, Shanghai, 201318 China; 3 Department of Urology, Huashan Hospital, Fudan University, Shanghai, China; 4 Reproductive medical center, Tongji Hospital, Tongji University School of Medicine, Shanghai, China

**Retraction Note:** ***J Exp Clin Cancer Res*** **39, 186 (2020)**

**https://doi.org/10.1186/s13046-020-01697-6**

The Editor-in-Chief has retracted this article at the authors' request. After publication, concerns were raised regarding the published images. Specifically:

- Fig. 4C appears to contain image overlap between different treatment groups and cell lines.
- Fig. 4F appears to contain overlapping areas with two articles on unrelated circRNAs that were under consideration within a similar time frame [1, 2].
- Figs. 6G and 7G appear to contain image overlap between different treatment groups and cell lines.

The authors have stated that the microscopy images in Fig. 4F were provided by a third party.

Due to the number of concerns with the images, the Editor-in-Chief and the authors no longer have confidence in the presented data.

All authors agree to this retraction.
